# The Brain-Specific Beta4 Subunit Downregulates BK Channel Cell Surface Expression

**DOI:** 10.1371/journal.pone.0033429

**Published:** 2012-03-16

**Authors:** Sonal Shruti, Joanna Urban-Ciecko, James A. Fitzpatrick, Robert Brenner, Marcel P. Bruchez, Alison L. Barth

**Affiliations:** 1 Department of Biological Sciences, Carnegie Mellon University, Pittsburgh, Pennsylvania, United States of America; 2 Molecular and Biosensor Imaging Center, Carnegie Mellon University, Pittsburgh, Pennsylvania, United States of America; 3 Department of Physiology, University of Texas Health Science Center at San Antonio, San Antonio, Texas, United States of America; University of Houston, United States of America

## Abstract

The large-conductance K^+^ channel (BK channel) can control neural excitability, and enhanced channel currents facilitate high firing rates in cortical neurons. The brain-specific auxiliary subunit β4 alters channel Ca^++^- and voltage-sensitivity, and β4 knock-out animals exhibit spontaneous seizures. Here we investigate β4's effect on BK channel trafficking to the plasma membrane. Using a novel genetic tag to track the cellular location of the pore-forming BKα subunit in living cells, we find that β4 expression profoundly reduces surface localization of BK channels via a C-terminal ER retention sequence. In hippocampal CA3 neurons from C57BL/6 mice with endogenously high β4 expression, whole-cell BK channel currents display none of the characteristic properties of BKα+β4 channels observed in heterologous cells. Finally, β4 knock-out animals exhibit a 2.5-fold increase in whole-cell BK channel current, indicating that β4 also regulates current magnitude in vivo. Thus, we propose that a major function of the brain-specific β4 subunit in CA3 neurons is control of surface trafficking.

## Introduction

The Ca^++^- and voltage-gated K^+^ channel BK (maxiK, slo, KCMNA1), broadly expressed throughout the CNS, modulates firing and neurotransmitter release [1], [2], [3], [4], [5], [6], [7]. BK channel mutations have been associated with familial epilepsy [8], [9], and BK channel antagonists can suppress cell-autonomous and network activity in vitro and prevent chemoconvulsant-induced seizures in vivo [4], [7], [10]. Furthermore, BK channel regulation has been linked to tolerance in alcoholism [11] and experience-dependent plasticity [12], [13], [14]. Thus, understanding the principles regulating BK channel function is relevant across many areas of contemporary neuroscience.

The BK channel is a tetramer of α subunits [15], [16] that assemble with an auxiliary β subunit in up to a 1∶1 stoichiometry [17], [18]. The four identified β subunit genes show tissue-specific expression (reviewed by [19]), where the most abundant CNS isoform is β4 [20], [21]. Coexpression of β4 in heterologous cells slows activation kinetics of BK channel currents [20], [22], [23], [24], generally increases the amount of Ca^++^ and depolarization required for channel gating [21], [23], [25] but see [26], and confers resistance to the specific peptide antagonists iberio- and charybdotoxin [25], [27]. Indeed, genetic knock-out of β4 results in larger BK channel currents gated by action potential (AP) firing and is associated with increased firing activity and spontaneous seizures in mice [1].

In contrast to the many investigations into how β4 influences the biophysical properties of BK channels, a role for this subunit in controlling the cellular location of BK channels has not been systematically investigated [28], although other β subunits can modulate trafficking of the channel [29], [30]. Regulated trafficking of BK channels in neurons is particularly interesting, as the number of endogenous channels at the plasma membrane may be small [31], [32], in the tens to hundreds range, in contrast to Na^+^ channels or glutamate receptors that are 100 to 1000-fold more densely distributed on the cell surface. Modest changes in the number of plasma membrane BK channels may profoundly influence the magnitude of whole-cell BK channel currents and, consequently, firing output and network excitability. Interestingly, although BK channels have been extensively studied in the CNS, few studies have detected BK channels with pharmacological or biophysical properties consistent with β4-containing channels [20], [21], [22], despite the broad expression of this subunit across the brain.

To investigate the role of β4 in regulating cell-surface trafficking of BK channels, we used a novel fluorogen activating protein (FAP) [33] to genetically tag the extracellular N-terminus of the BKα subunit. Cotransfection of tagged BKα with β4 in HEK-293 cells led to a 70% reduction in cell-surface channel fluorescence, assessed by application of a membrane–impermeant dye. In the presence of β4, BKα was sequestered in the ER, and mutation of a C-terminal ER retention/retrieval motif in β4 was sufficient to restore high levels of BKα at the plasma membrane.

How does β4 regulate BK channel currents in the CNS? Both BKα and β4 are highly expressed in CA3 pyramidal neurons. However, whole-cell BK channel currents from CA3 neurons in wild-type mice do not appear to contain β4, based upon their biophysical or pharmacological properties, consistent with a role for β4 in intracellular sequestration of the channel. Importantly, whole-cell BK channel currents in CA3 neurons from β4 knock-out mice are 2.5-fold larger than in wild-type or heterozygote animals. Taken together, our data indicate that the β4 subunit can regulate cell-surface BK channel localization.

## Materials and Methods

All animal experimental protocols were approved by Carnegie Mellon University's Institutional Animal Care and Use Committee (Animal welfare assurance number A3352-01).

### Cloning

Cortical and hippocampal tissues were collected from 3 C57BL/6 mice aged P14. RT-PCR was performed on RNA isolated from hippocampal tissue homogenates using gene-specific primers designed from annotated sequences (NCBI reference sequences NM_010610.2 for KCNMA1/BKα and NM_021452.1 for KCNMB4/β4), and PCR products were cloned and sequenced. The BKα variant isolated lacked the STREX exon. The β4 variant isolated was identical to published sequence (NM_021452.1 for KCNMB4/β4). The L5 FAP was affinity-matured using published procedures (Szent-Gyorgi 2008), and a novel variant (L91S) with a 5-fold improved quantum yield was isolated. The L5-FAP was fused in-frame to the BKα sequence beginning at the amino acids MDAL at the N-terminus. The FAP-BKα construct was synthesized according to the RT-PCR-derived BKα sequence in a pUC57 vector (Genscript Corporation), and the BKα and β4 constructs were subcloned into the pCS2+ using a CMV promoter to drive expression in mammalian cells. Mutant β4 constructs were created by replacing C- terminal lysines in the last six amino acids to alanines for the β4-Ala and by adding three serines to the C-terminal for the β4-polySer construct.

### Cell culture and transfection

HEK-293FT (Invitrogen) cells were grown in complete DMEM medium with FBS at 37°C at 5% CO_2_ and 75% humidity. Cells were seeded in 35 mm dishes and grown to ∼70% confluence for 24 hours. HEK-293 cells were transfected using Neofectin (Mid Atlantic Biolabs) according to manufacturer's specifications. One µg BKα DNA was transfected, and an equimolar amount of β4 was included where indicated. Cells were cultured for another 24 hours post transfection and were either imaged live at 37°C in a humidified chamber or fixed for immunocytochemistry.

### Live-cell imaging and analysis

Images were acquired on a LSM 510 Meta confocal microscope (Carl Zeiss). Immediately before imaging, media was replaced with complete DMEM without FBS with either cell impermeable MG-2p (100 nM) or cell permeable MG-ester dyes (100 nM). Cells were maintained at 37°C, 5% CO_2_ and 75% humidity throughout imaging. Fields for imaging and analysis were selected using only GFP fluorescence to identify transfected cells and were collected 3–10 minutes after dye addition.

Exported tiff files were analyzed in ImageJ. To determine surface fluorescence levels, mean cell membrane pixel intensities were determined as follows. A 250-pixel, circular ROI was placed on different regions of the plasma membrane of expressing cells (identified by both MG and GFP fluorescence) in a north-south-east-west orientation.

**Figure 1:**
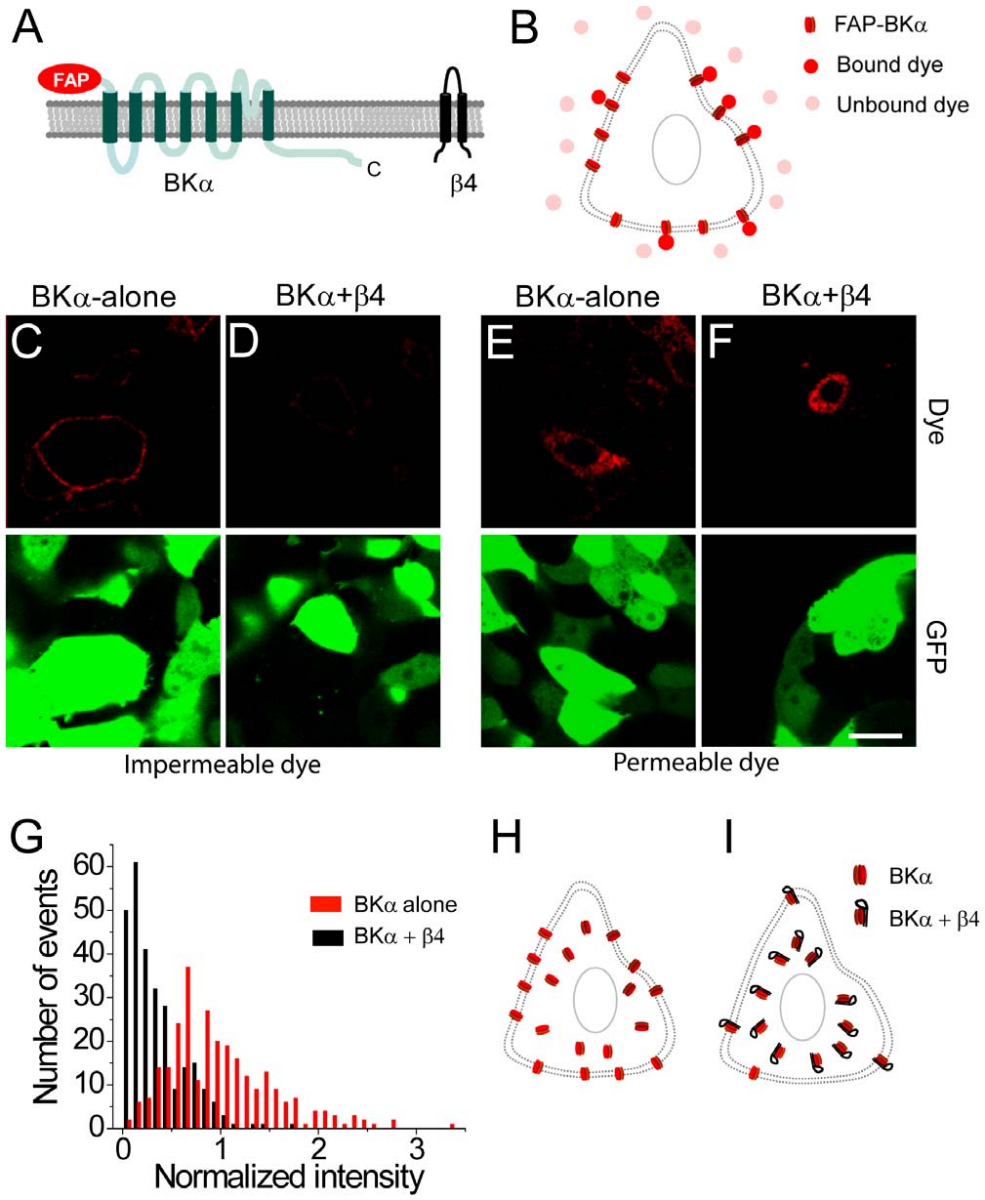
Co-expression of the β4 subunit reduces cell-surface trafficking of BK channels. (A) Membrane topology of the FAP-BKα and β4 proteins. The FAP tag is at the extracellular, N-terminus. The C-terminus of the BKα subunit is indicated. (B) Schematic of binding of cell-impermeable dye (pink) to the FAP results in significant increase in fluorescence (red). (C) Application of cell-impermeable dye labels only surface BKα channels in live HEK-293 cells co-transfected with FAP-BKα (red) and GFP (green). (D) Same as (C) but in cells co-transfected with β4, FAP-BKα, and GFP showing reduced surface expressed of the FAP-BKα. (E) Application of cell-permeable dye labels intracellular stores of channel in cells transfected with FAP-BKα (red) and GFP (green). (F) Same as (E) but in cells transfected with β4, FAP-BKα, and GFP. Scale bar = 20 µm (C–F). (G) Distribution of surface fluorescence intensity values after application of cell-impermeable dye in transfected cells for FAP-BKα (red bars) or FAP-BKα+β4 (black bars). (H–I) Proposed model for surface distribution of BK channels in the absence (H) or presence (I) of the β4 subunit.

Because transient transfections with multiple plasmids does not guarantee expression of all plasmids (for example, some cells may not have taken up the β4 plasmid), analysis was limited to cells that exhibited both GFP fluorescence as well as MG-2P surface signal. This method may have led to an overestimation of surface localization of FAP-tagged BKα in the BKα+β4 transfected cells, since some cells may not have expressed the β4 construct. Adjacent membranes from two transfected cells were rejected from analysis. ROIs for 2–4 cells were analyzed per image field, and 20 fields per transfection experiment were evaluated. Thus, ∼60 cells per condition were examined for each experiment. Experiments were repeated at least three times. Mean, minimum and maximum pixel intensities for each ROI were calculated, averaged for each cell, and then averaged across cells for each separate transfection. To account for potential differences in expression levels across different transfection experiments, all transfection datasets were normalized to the value of the mean surface fluorescence for the BKα-alone transfection for that specific experimental day. A histogram for the normalized fluorescence intensity values across all experimental days was generated (see Figure 1).

### *In situ* hybridization

Digoxigenin (DIG)-labeled probes were prepared using the Roche DIG RNA Labeling Kit. The β4 probe isolated from our RT-PCR experiments spanned the entire transcript. Brains were collected from C57BL/6 animals of either sex, perfused with 4% paraformaldehyde (PFA) and fixed overnight in PFA. The tissue was sunk in 30% sucrose and sectioned at 50 µm thickness on a cryostat. Sections were washed in PBS and MOPS buffer then placed in a hybridization solution overnight before incubating with the probe at 57°C for 5 days. Sections were then washed in MAB solution and blocked with 2% block (Roche) for 15 minutes at room temperature followed by overnight incubation with 1∶2000 alkaline phosphatase conjugated anti-DIG antibody (Roche) in MAB+2% block. Hybridization signal was detected with an alkaline phosphatase reaction at 37°C for several hours.

### Immunocytochemistry and analysis

Twenty-four hrs post-transfection, HEK-293 cells were fixed in 4% PFA+4% sucrose for five minutes at room temperature. Cells were washed with PBS and permeabilized using a solution containing 0.5% Triton X-100, 5% 1 M glycine and 5% normal goat serum for 30 minutes at room temperature. Appropriate primary antibodies were diluted in this blocking solution and incubated with the transfected cells for 1 hour at room temperature. After washing with PBS, the cells were incubated with the appropriate secondary antibody for 30 minutes at room temperature. For experiments with non-permeabilized labeling, Triton X-100 was replaced with PBS in all solutions. Primary antibodies used were anti-BKα (Neuromab) and anti-β4 (Neuromab for single labeling or Alomone labs for double-labeling) at a 1∶500 dilution. Secondary antibodies used were Alexa 488-conjugated goat anti-rabbit (Invitrogen) 1∶750, Alexa488-conjugated goat anti-mouse (Invitrogen) 1∶750, Alexa594-conjugated goat anti-mouse (Invitrogen) 1∶750 and Cy5-conjugated goat anti-mouse (Jackson Immuno Research) 1∶750.

**Figure 2:**
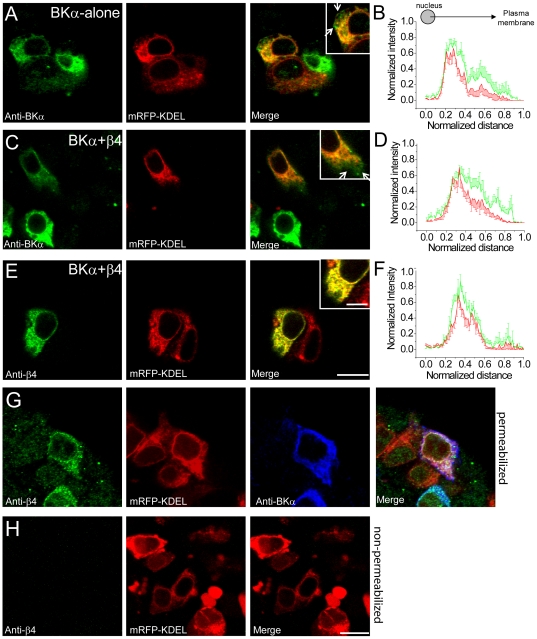
Immunofluorescence reveals that BKα and β4 are substantially localized to the ER and that β4 is difficult to detect on the cell surface. (A) Immunocytochemistry against fixed, permeabilized HEK-293 cells for the FAP-BKα subunit (anti-BKα: green) and the ER-marker mRFP-KDEL (intrinsic fluorescence; red) in cells transfected with BKα alone. Inset shows a cell with overlapping expression of BKα and mRFP-KDEL with arrows pointing to regions containing only BKα. (B) Quantitation of normalized intensity versus normalized distance from the cell nucleus for anti-BKα (green) and mRFP (red) in transfected cells. (C) As in (A) but for cells expressing BKα (green), β4 (unlabeled; not visualized) and mRFP-KDEL (red). (D) Same as (B) but in cells expressing BKα, β4 and mRFP-KDEL. (E) Localization of the β4 subunit (anti-β4: green) and mRFP- KDEL (intrinsic fluorescence; red) in cells expressing BKα (unlabeled; not visualized), β4 and mRFP-KDEL. Scale bar = 20 µm or 5 µm for inset. (F) As in (B), but for β4 and mRFP-KDEL. (G) Localization of the β4 subunit (anti-β4: green), mRFP-KDEL (intrinsic fluorescence; red) and BKα (anti-α: blue) in transfected, fixed HEK-293 cells expressing BKα, β4, and mRFP-KDEL. (H) Expression pattern of the β4 subunit (anti-β4: green) and mRFP- KDEL (intrinsic fluorescence; red) under non-permeabilized immunohistochemistry conditions. Note that immunohistochemical detection of cell-surface BKα is difficult because of an intracellular epitope for BKα. Scale bar = 10 µm (G,H).

Quantitation of overlapping expression for BKα or β4 with KDEL-mRFP in fixed cells was done as follows. A line was drawn from the center of the nucleus to a point just outside the cell membrane, for portions of the plasma membrane that were not abutting another cell, and pixel intensities in both channels along this line were recorded for 10 cells from two separate transfections. For all cells the values for each channel were normalized to their highest intensity, and distance was normalized to the maximum distance for that cell. The normalized values across all cells were averaged and plotted as shown in Figure 2.

### Electrophysiology

All electrophysiological experiments were performed as previously described [7]. The β4 knock-out mice used in these studies were inbred 5 generations to Jackson Labs C57BL/6J mice. β4 knock-out animals in the C57BL/6J genetic background were studied with EEG recordings and found to lack seizures (in contrast to the mixed 129/B6 background used in Brenner et al., 2005). In short, brains from P13–P15 C57BL/6 or β4 knock-out mice on a C57BL/6 background mice were sectioned coronally at 300 µm in a solution composed of (in mM): 110 choline chloride, 2.5KCl, 7 MgCl_2_, 1 NaH_2_PO_4_, 26.2 NaHCO_3_, 0.5CaCl_2_, 25 glucose, 11.6 sodium ascorbate and 3.1 sodium pyruvate. Slices were recovered in artificial cerebrospinal fluid (ACSF) composed of (in mM): 119 NaCl, 2.5 KCl, 1.3 MgSO_4_, 2.5 CaCl_2_, 1 NaH_2_PO_4_, 26.2 NaHCO_3_, 11 glucose equilibrated with 95/5% O_2_/CO_2_ at 34°C for 20 minutes and then maintained at room temperature. Recordings were performed at room temperature. Somata of CA3 pyramidal neurons in the hippocampus were targeted for whole-cell recording at room temperature with borosilicate glass electrodes with a resistance of 4–8 MΩ. Electrode internal solution was composed of (in mM): 116 potassium gluconate, 6 KCl, 8 NaCl, 4 MgATP, and 0.4 NaGTP, at pH 7.25–7.35, 290 mOsm. Free calcium was unbuffered to allow calcium accumulation during depolarization steps. Paxilline (Pax; 10 nM or 1 µm; Sigma) and Iberiotoxin (Ibtx; 50 nM; Sigma) were bath applied for at least 10 minutes to saturate channel block before further measurements.

To estimate the magnitude of steady-state BK channel currents in CA3 neurons, cells were held at −80 mV and a 40 ms prepulse of −40 mV followed by an 800 ms voltage step to +100 mV was applied before and after the application of paxilline or iberiotoxin. Currents from after drug application were subtracted from the pre-application currents to yield the paxilline-sensitive or iberiotoxin-sensitive BK channel current. The current from the voltage step was determined from an average of 4 sweeps. Capacitive current was subtracted for both the pre- and post-drug application currents by the P/4 method.

In comparison to recordings from heterologous cells, estimating BK channel currents in neurons is considerably more difficult. We are aware that our measurements may be dominated by BK channels that lie close to the soma, due to inadequate voltage clamp in the distal portions of the cell. However, because the critical comparisons for this study were of the steady-state currents isolated by two different drugs, and all other recording parameters were the same (cell type, cell age, input resistance), we expect that these uncontrolled variables should cancel out.

## Results

### β4 exerts a dominant negative effect on cell-surface trafficking of BK channels

Tracking the surface distribution of ion channels, a technically challenging issue in cellular neuroscience, has long been a subject of great interest. Although intrinsically fluorescent protein tags such as synaptophluorin have been used to examine surface expression of other membrane proteins, intracellular fluorescence is incompletely quenched at near-neutral pH in some intracellular compartments such as the ER [34]. Since many ion channels show enormous intracellular reservoirs, this fluorescence makes it difficult to resolve or quantitate lower levels of cell-surface staining. To bypass this, a FAP [33] was used to genetically tag the α subunit of the BK channel at the extracellular N-terminus [35] (Figure 1A). Upon small-molecule dye binding to the FAP, fluorescent output is enhanced 20,000-fold, providing outstanding signal-to-noise (Figure 1B). Because well-characterized antibodies against extracellular regions of BKα have not been generated, FAP-tagging of the protein enabled direct comparison of cell-surface channel localization using a cell-impermeable dye, malachite green diethylene glycol (MG-2p; [33]), in the presence and absence of β4. Furthermore, this analysis could be carried out in living cells under normal growth and temperature conditions. This is particularly important, because channel trafficking can be acutely altered by temperature and mechanical stress [29], [30], [36].

The genes encoding BKα and β4 were isolated from mouse brain tissue, and cloned into a mammalian expression vector. Sequences matched the BKα STREX-lacking isoform [37] and the canonical β4 transcript (Figures S1, S2). The L5 FAP tag was fused in frame to the N-terminus of BKα, at the amino acid sequence beginning MDALIIPV. In HEK-293 cells transfected with FAP-tagged BKα alone, application of the impermeable MG-2p dye revealed a clear outline of the plasma membrane (Figure 1C). Cotransfection of FAP-BKα with unlabeled β4 revealed a marked reduction in cell-surface fluorescence in the presence of MG-2p dye (Figure 1D). This was not due to suppression of BKα-protein in cotransfected cells, since application of a membrane-permeable MG-ester dye revealed FAP-BKα in intracellular compartments under both transfection conditions (Figure 1E, F).

**Figure 3:**
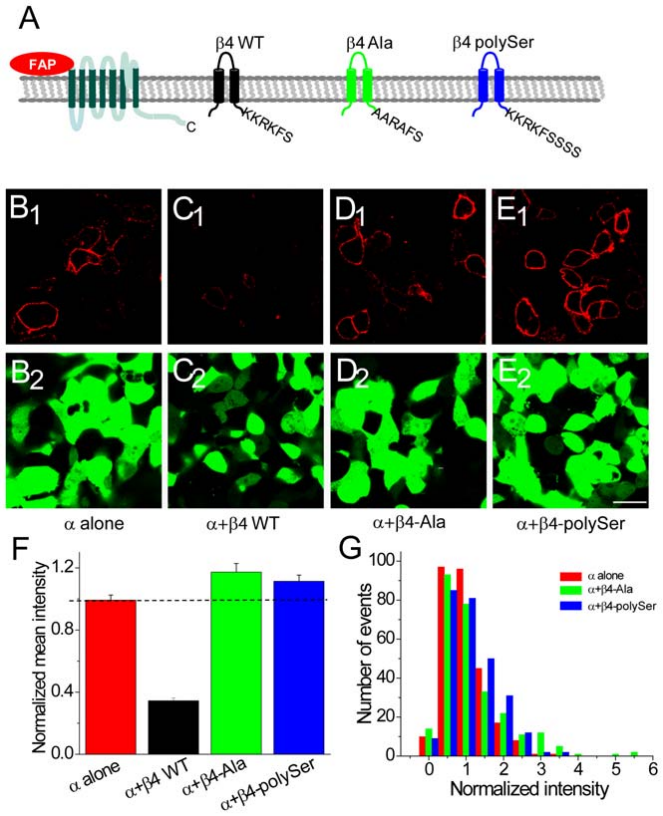
An ER-retention/retrieval sequence at the C-terminus of β4 inhibits the surface expression of BKα and β4. (A) Schematic of the mutant β4 constructs showing the C-terminal amino acids. (B) Fluorescence from binding of cell-impermeable dye to cell-surface BKα channels in cells transfected with FAP-BKα (red) and GFP (green). (C) Same as (B) but in cells transfected with FAP-BKα and wild-type β4. (D) Same as (B) but in cells transfected with FAP-BKα and β4-Ala. (E) Same as (B) but in cells transfected with FAP-BKα and β4-polySer. Scale bar = 20 µm (B–E). (F) Mean intensity of surface fluorescence for β4 constructs normalized to fluorescence from cells transfected with FAP-BKα alone. (G) Histogram showing distribution of fluorescence intensity in cells transfected with FAP-BKα alone (red), FAP-BKα+β4-Ala (green) and FAP-BKα+β4-polySer (blue). Scale bar: 20 µm (B–E).

Quantitative analysis of cell-surface fluorescence was carried out for FAP-BKα-alone and FAP-BKα+β4 transfected cells. Because HEK-293 cells can show a range of values for surface fluorescence, a phenomenon that is likely to depend upon different levels of gene expression in the transfected cells, a histogram was constructed for fluorescence values across multiple cells for each transfection condition. A significant difference in the across-cell distribution of fluorescence intensity was observed for the two experimental groups (Figure 1G; n = 54 regions of interest (ROIs) for each condition, averaged over five independent transfections, 270 ROIs total, p<10^−3^ by two-sample K-S test). Overall, the presence of β4 induced a nearly 70% reduction in mean surface fluorescence (normalized mean surface intensity, FAP-BKα-alone, 1±0.03, versus FAP-BKα+β4, 0.34±0.02, p<10^−5^ by paired t-test; see also Figure 3). Similar intracellular but different cell-surface expression of BKα in the absence/presence of β4 are schematized in Figure 1H,I.

As an additional method to evaluate β4-regulated surface expression of BKα, dual-label immunohistochemistry on transfected, fixed HEK-293 cells was performed and analyzed by confocal microscopy of a thin z-section through the cell (Figure S3). These experiments took advantage of small differences in the level of β4 expression in different transfected cells and allowed comparison of surface to internal BKα on a cell-by-cell basis. Surface BKα was tightly coupled to the amount of β4 expressed, with cells expressing little β4 showing the greatest surface∶internal ratio. Conversely, cells that expressed high levels of β4 exhibited the lowest ratio of surface: internal BKα immunofluorescence (Figure S3A–F). This conclusion was further supported by live cell imaging experiments using spectrally-distinct cell-permeable and impermeable dyes. In BKα+β4 transfected cells, surface labeling of the FAP-tagged BKα was notably lower compared to the BKα-alone although intracellular levels of BKα appeared grossly similar (data not shown).

### β4 concentrates BK channels in the ER

β4 expression might reduce surface BKα levels by facilitating degradation of the pore-forming channel, or by interacting with BKα subunits to reduce cell-surface trafficking. To determine whether BKα and β4 are concentrated in the same intracellular compartment and to identify this structure, HEK-293 cells were co-transfected with FAP-BKα, β4, and monomeric RFP (mRFP) tagged with an N-terminal prolactin signal sequence and a C-terminal sequence (KDEL) that leads to protein retention in the endoplasmic reticulum (ER; mRFP-KDEL; gift of T. Lee and E.L. Snapp; [38]). Importantly, untransfected HEK-293 cells exhibited no BKα or β4 immunoreactivity, indicating that these cells do not express endogenous BK channel subunits that might alter trafficking results [39] (and data not shown).

In FAP-BKα and mRFP-KDEL transfected and fixed cells, clear colocalization of immunolabeled BKα and mRFP was observed, indicating that the bulk of BKα subunits reside in the ER (Figure 2A). This is similar to what has been observed for other K^+^ channel proteins that also show large intracellular reserves [40], [41], [42]. By immunohistochemistry, BKα signal was not pronounced at the margins of the cell, highlighting the benefits of the FAP tag that allows visualization of cell-surface protein without spectral contamination from intracellular stores.

In FAP-BKα+β4-expressing cells, BKα immunolabeling showed similar overlap with mRFP-KDEL (Figure 2C). However, in both FAP-BKα-alone and β4 cotransfected cells, some BKα could be observed outside of the mRFP-KDEL ER compartment, indicating that at least some fraction of the BKα subunit may escape the ER (Figure 2B,D; both distributions are significantly different, p<0.001 by K-S test).

β4 immunostaining in fixed transfected cells confirmed both β4 subunit expression and β4 colocalization with BKα and KDEL-mRFP (Figure 2E–G). Localization analysis indicated that β4 and mRFP-KDEL distributions were not significantly different (Figure 2F, p>0.1), suggesting that the β4 subunit is largely retained within the ER. Based upon the finding that β4 co-expression is sufficient to reduce surface FAP-BKα, as well as that immunofluorescence for both proteins show substantial overlap with mRFP-KDEL and with each other, we propose that β4 directly interacts with the BKα subunit to retain the channel complex in the ER.

Since the epitope for the β4 antibody is within the extracellular loop of the two-transmembrane domain protein (see Figure 1A), surface localization of β4 can be determined by immunocytochemistry in non-permeabilized cells. Under these conditions, β4 immunoreactivity was not observed at the cell surface (Figure 2H) even though significant β4 expression can be seen after permeabilization (Figure 2G). These data show that although transfected HEK-293 cells indeed express β4 protein, this β4 protein is undetectable at the plasma membrane.

### Identification of an ER retention motif in β4

The amino acid determinants of proteins that reside in the ER have been well-defined [41], [43], [44], [45]. Canonical amino acid motifs for ER retention/retrieval of both lumenal and transmembrane proteins are KDEL or KKXX at the carboxy (C)-terminus (where XX are the last two amino acids of the protein) respectively. Although β4 lacks either of these canonical motifs, the last six amino acids, KKRKFS, may also serve as an ER retention sequence under some conditions [44] (Figure 3A).

To test whether these amino acid residues in β4 were sufficient to exclude co-expressed FAP-tagged BKα from the plasma membrane, these lysine residues were mutated to alanines (β4-Ala; Figures 3A, S2). Because it has also been demonstrated that the KKXX motif must be at the C-terminus of the protein [44], we also investigated whether addition of three amino acids (serines) to the C-terminus would disrupt the ER retention/retrieval function of β4 (β4-polySer; Figures 3A, S2).

Cotransfection of FAP-BKα with β4-Ala or FAP-BKα with β4-polySer, followed by application of the impermeable MG-2p dye, exhibited robust surface fluorescence for both co-transfected β4 mutants (Figure 3B–E). Quantitation of surface fluorescence intensity across cells indicated that mean surface fluorescence was similar or even greater than FAP-BKα-only transfected cells (normalized mean surface intensity: β4-Ala 1.17±0.06, β4-polySer 1.12±0.04; significantly different from BKα-alone by ANOVA; n = 54 ROIs per transfection experiment over 5 experiments total for each mutant; Figure 3F). In addition, the distribution of FAP-BKα fluorescence surface intensity values for the two mutant β4 proteins was fully overlapping with that observed for FAP-BKα-alone transfected cells (p = 0.10 by two-sample K-S test for β4-Ala and p = 0.13 for β4-polyser versus BKα alone; Figure 3G). Thus, the β4 C-terminal motif KKRKFS is required for ER retention of the BKα channel subunit in transfected HEK-293 cells.

**Figure 4:**
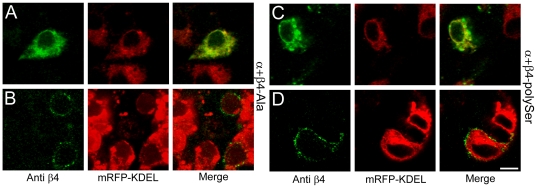
Mutation of the C-terminal ER-retention/retrieval sequence in β4 liberates β4 to the surface. (A) Expression pattern of the cotransfected FAP-BKα (unlabeled; not visualized), β4-Ala mutant (anti-β4 immunocytochemistry: green) and mRFP- KDEL (intrinsic fluorescence; red) in fixed, permeabilized HEK-293 cells. (B) Same as (A) but under non-permeabilized conditions. (C) Same as for (A) but for β4-polySer mutant under permeabilized conditions. (D) Same as (C) but under non-permeabilized conditions. Scale bar = 10 µm (A–D).

An alternate explanation for the increased surface localization of BKα is that mutant β4 constructs are not efficiently expressed in transfected cells. To verify that the β4-Ala and β4-polySer constructs are present in cells expressing BKα, β4-immunocytochemistry was carried out in the FAP-BKα and mutant β4 transfected HEK-293 cells. Strong intracellular immunoreactivity for both β4-Ala (Figure 4A) and β4-polySer was observed, indicating that these mutations do not markedly suppress β4 protein levels.

Immunocytochemistry using a β4 antibody in non-permeabilized cells revealed robust cell-surface staining for both β4-Ala and β4-polySer cotransfections with FAP-BKα (Figure 4B,D). This demonstrates that our experimental conditions are sufficient to detect not just FAP-tagged BKα, but also β4 protein, at the cell surface. The presence of both FAP-tagged BKα and mutant β4 at the cell surface suggest that modifications of the C-terminal sequence of β4 are sufficient to liberate BKα+β4 channels from the ER.

### β4-containing channels have a minor contribution to whole-cell BK channel currents in hippocampal neurons

Although many trafficking studies are carried out in heterologous cells, protein overexpression may alter steady-state distributions of channels. A more appropriate test of whether β4-containing BK channels are present and functional at the cell surface is to examine BK channel properties in CNS neurons that endogenously express high levels of β4. Because the majority of BK channels in neurons, as in HEK-293 cells, reside in intracellular stores [46], cell-surface immunolocalization of BK channels in brain tissue is difficult to resolve. However, BK channel currents can be electrophysiologically isolated using whole-cell patch clamp recording, a direct method to analyze BK channel function in living neurons.

**Figure 5:**
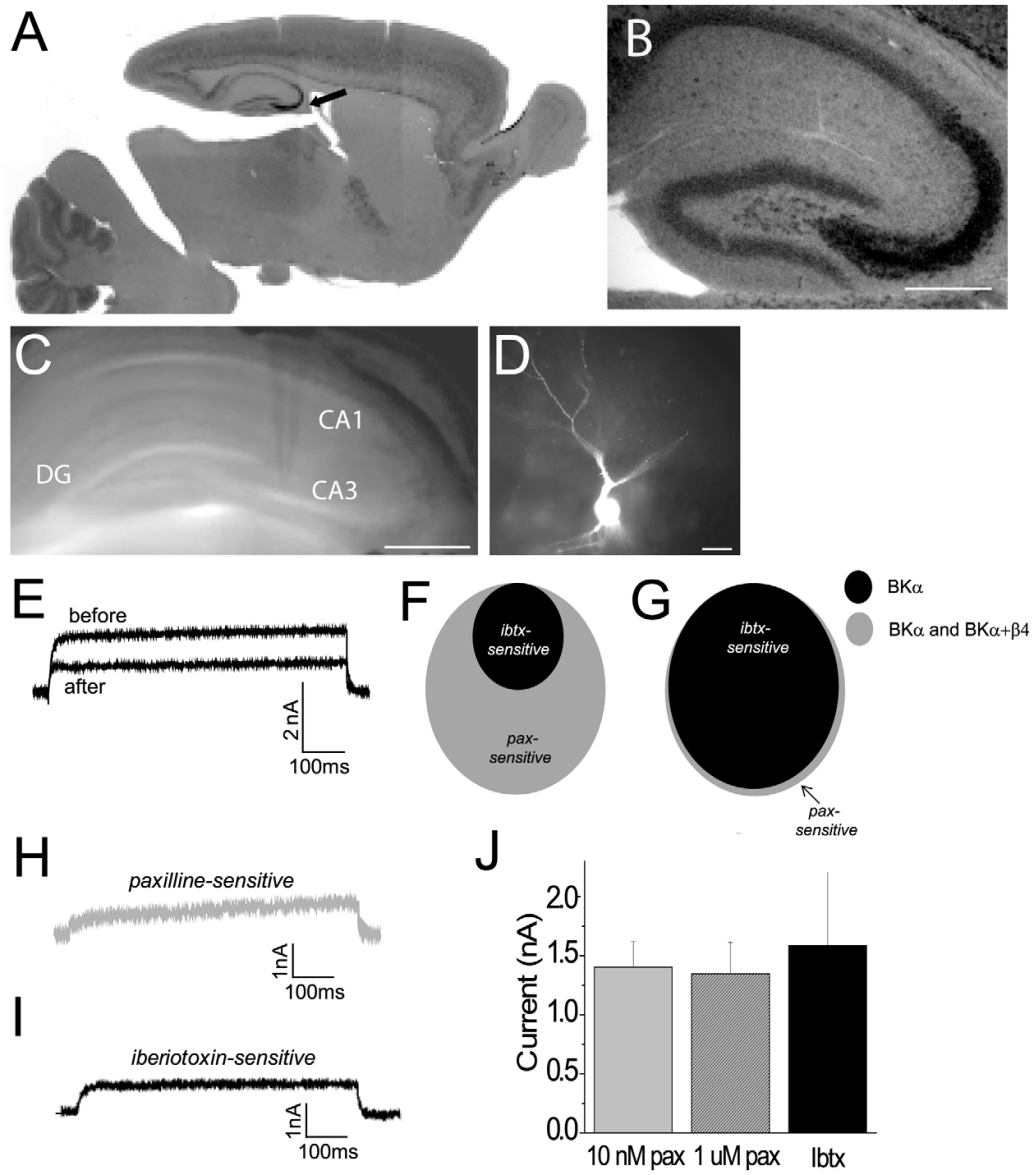
β4-containing BK channels do not contribute to whole-cell BK channel currents. (A) In situ hybridization showing regions of β4 expression in a mouse brain. Arrow points to high levels of expression in the CA3. (B) Hippocampal area CA3 shows robust β4 expression. Scale bar = 200 µm. (C) Bright field image showing a whole cell electrode in the CA3. Scale bar = 500 µm. (D) Fluorescent image of a CA3 pyramidal cell filled with Alexa fluor 568 after whole cell patch clamp recording. Scale bar = 50 µm. (E) Overlaid traces from a representative cell showing total potassium current and the residual potassium current after application of a BK channel blocker. (F) Schematic overlap between paxilline- and iberiotoxin-sensitive currents when β4-containing BK channels contribute to whole-cell current. (G) Schematic overlap between paxilline- and iberiotoxin-sensitive currents when β4 containing BK channels do not contribute to whole-cell current. (H) Example paxilline-sensitive BK channel current from a representative cell. (I) Example iberiotoxin-sensitive BK channel current from a representative cell. (J) Quantitation of mean paxilline (pax)- and iberiotoxin (ibtx)-sensitive currents from CA3 pyramidal cells.

To identify brain areas that express elevated β4 in control tissue, in situ hybridization using a β4 anti-sense probe was performed. Hybridization in hippocampal area CA3 was substantially greater than in almost any other brain area (Figure 5A, B). If β4-containing BK channels at the cell-surface play a major role in regulating neuronal BK currents, we reasoned that this would be prominent in these neurons.

To probe for the presence of BKα+β4-containing channels, whole-cell patch-clamp recordings from CA3 pyramidal neurons were carried out and BK channel currents were identified using a pharmacological subtraction method (Figure 5E). The pan-BK antagonist paxilline, which blocks all BK channels, was used to isolate the whole-cell BK current, and this value was compared to the amplitude of the iberiotoxin-sensitive current. If the majority of current is carried by BKα+β4 channels, we reasoned that the amplitude of the iberiotoxin-sensitive current would be significantly smaller than the paxilline-sensitive current (schematized in Figure 5F). Alternatively, if there was little or no current carried by BKα+β4 channels, there should be no difference between the amplitude of the paxilline-sensitive and the iberiotoxin-sensitive current (schematized in Figure 5G).

Whole-cell K^+^ currents were isolated before and after antagonist application and subtracted offline. Because β4-containing channels are slowly activating and may take hundreds of milliseconds to reach peak current [20], [21], [23], current amplitude was calculated at the end of an 800 ms pulse from −80 to +100 mV (Figure 5H–J).

Using the pan-BK channel antagonist paxilline, the magnitude of steady-state BK channel current was 1.40±0.22 nA (10 nM paxilline; n = 7 cells; Figure 5H). Although these experiments typically used a concentration of paxilline that reflects the low IC_50_ established for this drug [47], [48], similar current amplitudes were also obtained using drug concentrations that were 100-fold higher (1 µM paxilline 1.35±0.26 nA, n = 6 cells; p = 0.9 versus 10 nM paxilline; Figure 5H,J). Thus, it is unlikely that we systematically underestimated the amplitude of BK channel currents.

Surprisingly, the magnitude of iberiotoxin-sensitive BK channel currents was nearly identical to that obtained using the pan-BK channel antagonist paxilline (50 nM iberiotoxin; 1.59±0.62 nA, n = 6 cells; p = 0.8 versus 10 nM paxilline; Figure 5I,J). These data suggest that there is little contribution of β4-containing BK channels to whole-cell BK channel currents in CA3 pyramidal neurons (Figure 5G). It is alternatively possible that BK channels in CA3 neurons may contain β4 with a reduced stoichiometry that is sensitive to iberiotoxin blockade.

The influence of β4 on the biophysical properties of BK channel currents has been well-studied [1], [20], [21], [22], [23], [24], [25], [26], [27]. In HEK-293 cells, where voltage clamp can be excellent, β4 typically slows the activation kinetics (τ) of BK currents [1], [20], [23], [26]. Indeed, these effects of β4 have been postulated to strongly influence channel function in vivo [1]. Estimating τ for BK channel currents in neurons is problematic, in part because voltage clamp is imperfect in these cells. Activation time constants for iberiotoxin and paxilline-sensitive currents were comparable (τ = 2.01±0.56 ms for 10 nM paxilline; 2.89±1.22 ms for 50 nM iberiotoxin; p = 0.29).

Some previous studies, including those where recordings from CNS neurons were carried out, have identified iberiotoxin-resistant BK channel currents. For example, in neurons from the dentate gyrus, single-channel recordings clearly have shown iberiotoxin-resistant BK channels from membrane patches pulled from the cell soma [1]. To resolve this contradiction and determine whether our inability to observe β4-containing channels might be due to our whole-cell recording approach, we also compared paxilline and iberiotoxin-sensitive whole-cell BK channel currents from dentate gyrus neurons. The magnitude of the iberiotoxin-sensitive current was more than 10-fold smaller than the paxilline-sensitive current (iberiotoxin 0.11+0.2 nA; n = 5 cells versus paxilline 1.6+0.06 nA; n = 3 cells). The activation kinetics of the pharmacologically isolated BK channel current was similar between the iberiotoxin and paxilline-sensitive current, suggesting that this measurement in neurons is an unreliable indicator of the molecular composition of the channel (τ for iberitoxin-sensitive current 8.41+1.8 ms versus paxilline 7.26+1.38 ms). These data show that the pronounced effect that β4 has on BK channel trafficking can be influenced by neuron cell type.

### BK channel currents are larger in β4 knock-out animals

**Figure 6:**
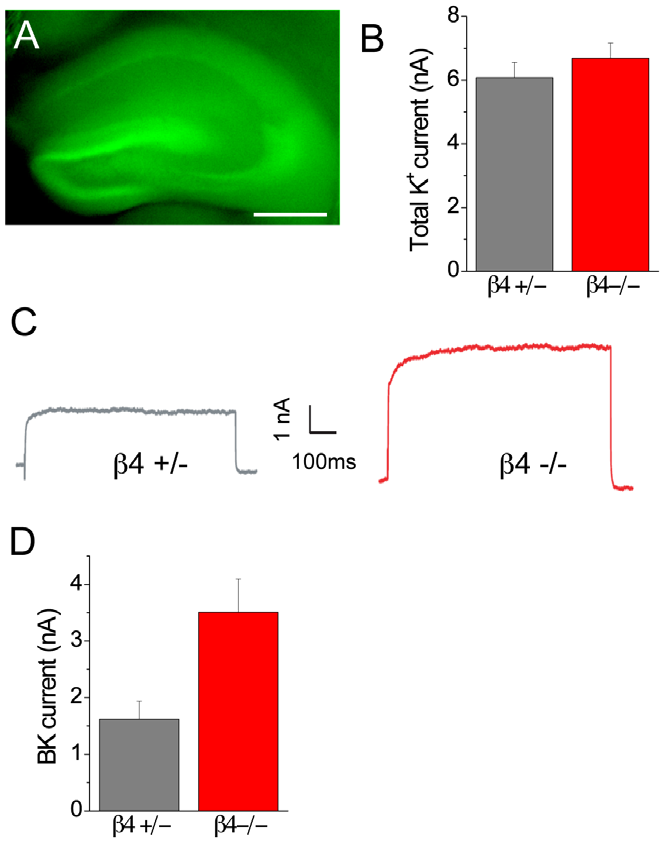
Genetic ablation of β4 results in larger BK channel currents in CA3 pyramidal neurons. (A) Fluorescent image of a living slice from a β4−/− knock-out mouse showing GFP-signal in hippocampus. Scale bar: 500 µm. (B) Mean total potassium current from β4 heterozygote (β4+/−) and β4 knock-out (β4−/−) mice. (C) Example paxilline-sensitive BK channel current from representative CA3 neurons in β4+/− and β4−/− mice. (D) Mean amplitude of paxilline-sensitive BK channel current in β4+/− and β4−/− mice.

A critical test of the hypothesis that β4 can regulate whole-cell BK channel current is to examine the amplitude of BK current in CA3 neurons where no β4 is expressed. Consistent with our in situ hybridization results, hippocampal area CA3 showed strong expression of the GFP reporter that had been knocked-in to the β4 expression locus (Figure 6A). Pharmacological isolation of whole-cell BK channel current revealed that CA3 neurons in β4 knock-out animals showed a highly significant, more than 2.5-fold increase in BK channel current amplitude compared to heterozygote littermates (paxilline-sensitive current in β4 +/− mice: 1.62±0.32 nA; n = 11 cells versus β4 −/− mice 3.51±0.59 nA; n = 12 cells; p = 0.01, Figure 6C,D). BK channel current amplitude in heterozygote animals was comparable to values from wild-type animals (see Figure 5J).

We saw no effect on the activation kinetics for the pharmacologically subtracted currents, which were comparable between the heterozygote and knock-out groups (τ = 2.20±0.46 in β4 +/− mice versus 1.61±0.32 in β4 −/− animals, p>0.1). These data are in concordance with the negative-regulatory function of β4 on BK channel surface expression that were obtained via live-cell imaging after channel overexpression in heterologous cells.

## Discussion

BK channels, because of their large single-channel conductance and co-activation by depolarization and intracellular Ca^++^, play an important role in regulating neuronal firing. BK channels disproportionately contribute to neuronal whole-cell K^+^ channel currents, where they can carry more than half the total voltage-gated K^+^ current. We have shown that expression of cell-surface BK channels is controlled by the presence of the brain-specific β4 subunit. Using a novel FAP to track the location of tagged BK channels at the cell surface and cytoplasm, we find that coexpression of β4 significantly reduces BKα channel protein at the plasma membrane, a function that is dependent upon a C-terminal ER retention/retrieval motif.

In CA3 neurons, which exhibit the highest levels of β4 expression compared to almost any neurons within the rodent CNS, pharmacologically-isolated whole-cell BK channel currents display none of the expected characteristics for BKα+ β4 channels, suggesting that these channels may not be present at the cell surface. Furthermore, genetic ablation of the β4 subunit was sufficient to significantly increase whole-cell BK channel currents in knock-out animals. Thus, we propose that an important function of the CNS-specific BK channel accessory subunit β4 in CA3 neurons is control of cell-surface trafficking of the BK channel complex.

These studies employed a FAP tag to track the location of BK channels in living cells. This methodology has significant advantages compared to more standard techniques, such as GFP protein tags, where the surface fluorescence signal can be overwhelmed by the much larger intracellular stores of channel protein. Although the GFP-based Phluorin [49], a fluorescence protein tag whose signal is quenched in acidic cell compartments, has been useful for studying vesicle fusion [50], [51], the nearly neutral pH of the ER has been associated with breakthrough fluorescence that complicates analysis. Advantages of the FAP system are use of membrane permeable and impermeable dyes, high signal-to-noise due to low fluorescence of unbound dye, and the potential to carry out real-time imaging experiments. Since unbound dye has essentially no fluorescence, a specific signal is generated without washing off excess dye, a property that will enable imaging in more complex tissue environments. Additionally, because the fluorescence signal from a single- dye-FAP interaction can be so bright (5 to 20-fold brighter than GFP), this technology may be particularly well-suited to studying the localization and dynamics of individual molecules, such as single BK channels at the plasma membrane.

Much experimental effort has gone into understanding the biophysical consequences of β4 on BK channel function, with little attention to how this auxiliary subunit can control channel localization. Using live-cell imaging and immunolocalization in heterologous cells, as well as electrophysiological measurements in CA3 neurons that express high levels of β4, we find that β4 expression is associated with a dramatic reduction of BK channels at the cell surface. In addition, we find that genetic ablation of β4 is sufficient to significantly enhance whole-cell BK channel currents. This may explain the paradoxically broad expression of β4 in the CNS (Figure 6 and [20], [21], [22]) despite iberiotoxin-pharmocology indicating that BK channels lack β4 in many neurons [52], [53], [54], [55], [56], [57]. Taken together, our results suggest that the effect of β4 on channel function may be much more indirect than previously imagined.

Nevertheless, a few studies report the presence of iberiotoxin-insensitive BK currents in the CNS, specifically in posterior pituitary nerve terminals and dentate gyrus granule neuronal soma and their mossy fiber terminals [1], [2], [58], [59]. Further, the β4 subunit was found to promote surface expression of the related slo3 channel in Xenopus oocytes [60]. How can the present findings be resolved with this? Our comparison of iberiotoxin and paxilline-sensitive currents in dentate gyrus neurons indicates that in some neurons, β4 is not sufficient to reduce cell-surface channel expression. Thus, there may be additional factors, including activation of signaling pathways, which can regulate channel localization in a cell-type specific manner.

Some studies have also suggested that the β4 subunit may regulate subcellular distribution of the BK channels into axonal or dendritic compartments [6], [59], [61], [62]. Because of the limitations of voltage clamp in neurons (i.e. poor voltage control in the distal processes of the cell), such β4-containing channels might be hard to detect in CA3 neurons. It is also possible that BK channels can be redistributed to the plasma membrane under some circumstances, for example, following mechanical dissociation of cells prior to analysis [36], [62], [63], [64], or during periods of high firing. Redistribution could occur over very short time intervals (100 s of ms), during firing bursts, or might be regulated over the course of many minutes. Such regulation has been observed for K_v_2.1 type K^+^ channels [46]. Our finding that β4 expression is associated with the regulation of surface levels of BK channels suggests a new mechanism for dynamic control of channel activity.

A caveat of the present study is that direct association of the BKα subunit with β4 was not directly demonstrated in transfected cells. However, the rescue of surface expression with co-transfection of the β4 mutant construct suggests that there is some interaction between the two proteins. Furthermore, our preliminary recordings from transfected HEK-293 cells indicate that the increase in surface expression observed in BKα+β4-Ala expressing cells is linked to iberiotoxin-resistant whole-cell currents. Further studies will be required to unambiguously demonstrate the interaction of BKα and β4 in our experimental preparation.

Enhanced BK channel currents have been linked to neuronal hyperexcitability and epilepsy in cortical neurons [1], [7], [9], [65]. Indeed, BK channel antagonists can reduce bursting in abnormally active tissue [4], [7] and have a profound anticonvulsant effect in vivo [10]. The large contribution of BK channels to the total K^+^ channel current in CA3 neurons suggests that these channels may play a particularly important role in controlling the excitability of these cells.

The data presented here characterize the detailed molecular mechanisms that control cellular BK channel trafficking. We have identified a new role for the BK β4 channel subunit – ER retention – as well as the specific amino acid residues that are necessary to direct this function. Additionally, we have shown that BK channels in CA3 neurons are not associated with the pharmacological and biophysical hallmarks of β4-containing channels as described in previous studies using heterologous cells, and that the absence of β4 is sufficient to significantly enhance whole-cell BK channel current. Because these studies were carried out in CNS neurons in acute brain slices, these findings may be particularly relevant to channel function *in vivo*. The dynamic regulation of endogenous BK channel localization in neurons is an exciting avenue for future investigations.

## Supporting Information

Figure S1 **Sequence of BKα and FAP L5.** (A) Sequence of BKα; upper case letters in green: Start codon; Upper case letters in red: Stop codon; Bold italicized text: Exon start site; Bold text in red: ALCOREX/exon 24; Black arrows show position of primers used in cloning BKα. (B) DNA sequence of FAP L5. (C) Amino acid sequence of FAP L5. The L91S mutation is in red.
(TIF)

Figure S2 **Sequence of wild-type and mutant β4.** Bold text in red: Changes from wild type β4 sequence. Black arrows show position of primers used in cloning β4.
(TIF)

Figure S3 **β4 reduces surface expression of BK channel α subunit.** (A–C) Immunocytochemistry on fixed, permeabilized HEK-293 cell expressing FAP-BK α+β4 subunits. (A) Anti-BKα immunocytochemistry. (B) Anti-β4 immunocytochemistry for the same cell. (C) Merge of (A) in red and (B) in green. Scale bar = 10 µm (A–C). (D) Zoom of region within the red box in (A). The area within D1 and D2 are example ROIs used to calculate the intensity of surface (D1) and internal (D2) BKα expression, respectively, in a transfected cell. (E) Zoom of region within the red box in (B). The area within E1 is an example ROI used to calculate the intensity of β4 in a transfected cell. Scale bar = 5 µm (D–E). (F) Graph shows the mean BKα surface to internal ratio (i.e., D1/D2) to β4 expression (i.e., E1) for individual cells (30 ROIs per cell, n = 31 cells).
(TIF)
