# Correction: The Brain-Specific Beta4 Subunit Downregulates BK Channel Cell Surface Expression

**DOI:** 10.1371/annotation/64524ba8-f739-4fa8-93bf-862b160dca5c

**Published:** 2012-05-07

**Authors:** Sonal Shruti, Joanna Urban-Ciecko, James A. Fitzpatrick, Robert Brenner, Marcel P. Bruchez, Alison L. Barth

The following details were mistakenly excluded from the published Funding statement: R.B was supported by NIH NS052574. Additional support in the early phases of this project was provided by a grant from the Milken Family Foundation/Epilepsy Research Foundation to A.L.B. 

