# Supplementary figures and images for: The Brain-Specific Beta4 Subunit Downregulates BK Channel Cell Surface Expression
Source: PLoS One. 2012 Mar 16;7(3):e33429. doi: 10.1371/journal.pone.0033429 (PMC3306404; doi:10.1371/journal.pone.0033429)

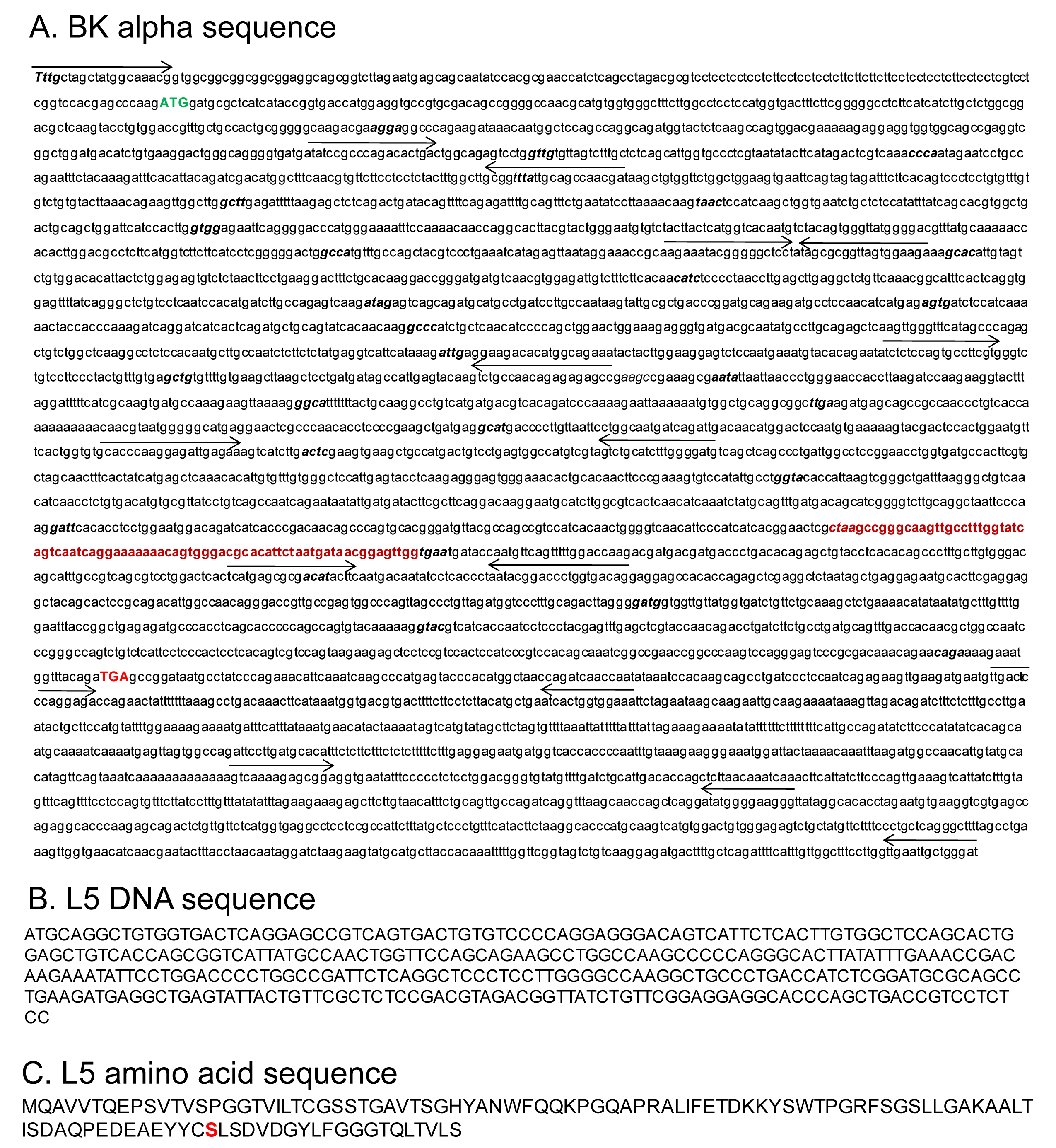

Supplement: Figure S1 — Sequence of BKα and FAP L5. (A) Sequence of BKα; upper case letters in green: Start codon; Upper case letters in red: Stop codon; Bold italicized text: Exon start site; Bold text in red: ALCOREX/exon 24; Black arrows show position of primers used in cloning BKα. (B) DNA sequence of FAP L5. (C) Amino acid sequence of FAP L5. The L91S mutation is in red. (TIF) [file pone.0033429.s001.tif]

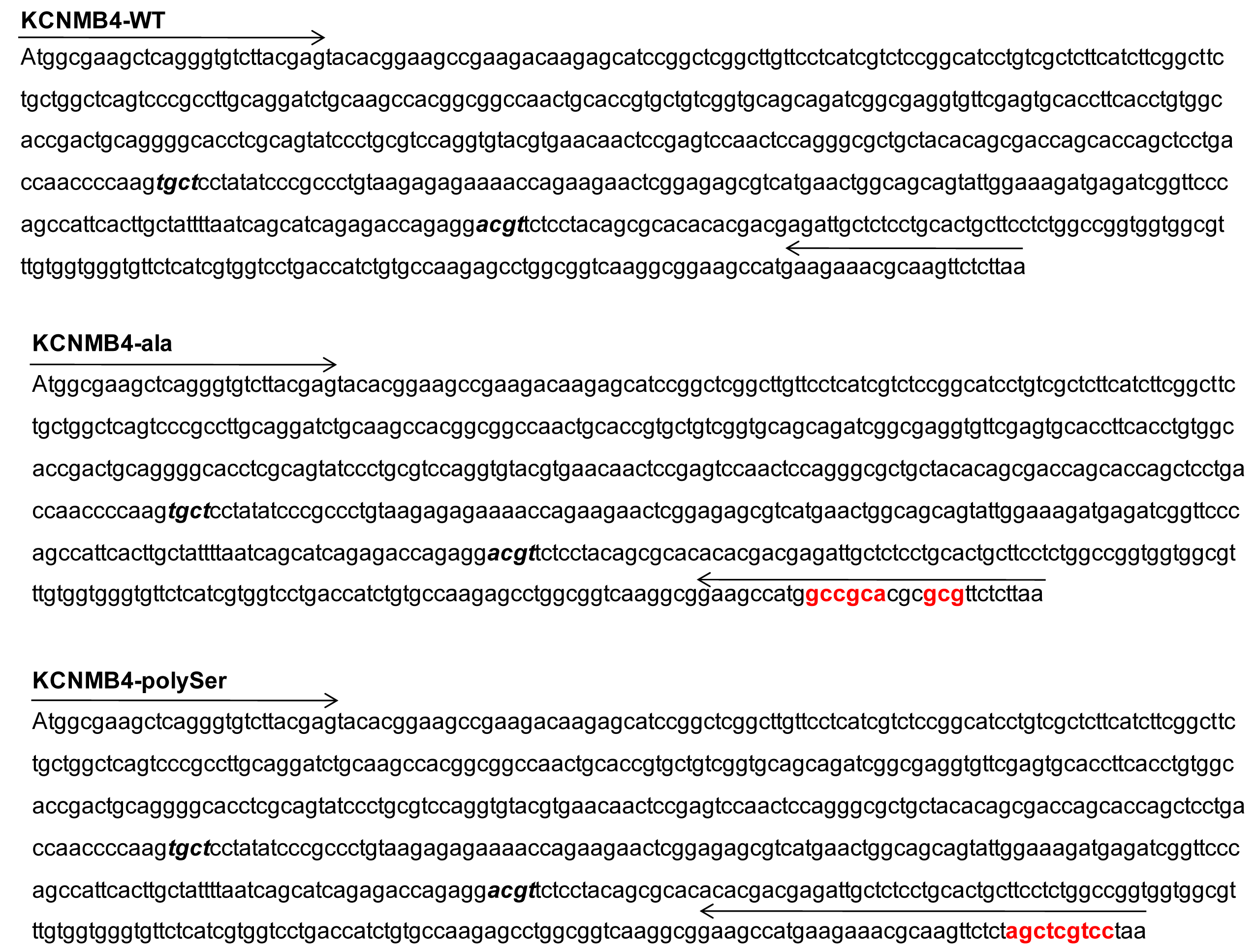

Supplement: Figure S2 — Sequence of wild-type and mutant β4. Bold text in red: Changes from wild type β4 sequence. Black arrows show position of primers used in cloning β4. (TIF) [file pone.0033429.s002.tif]

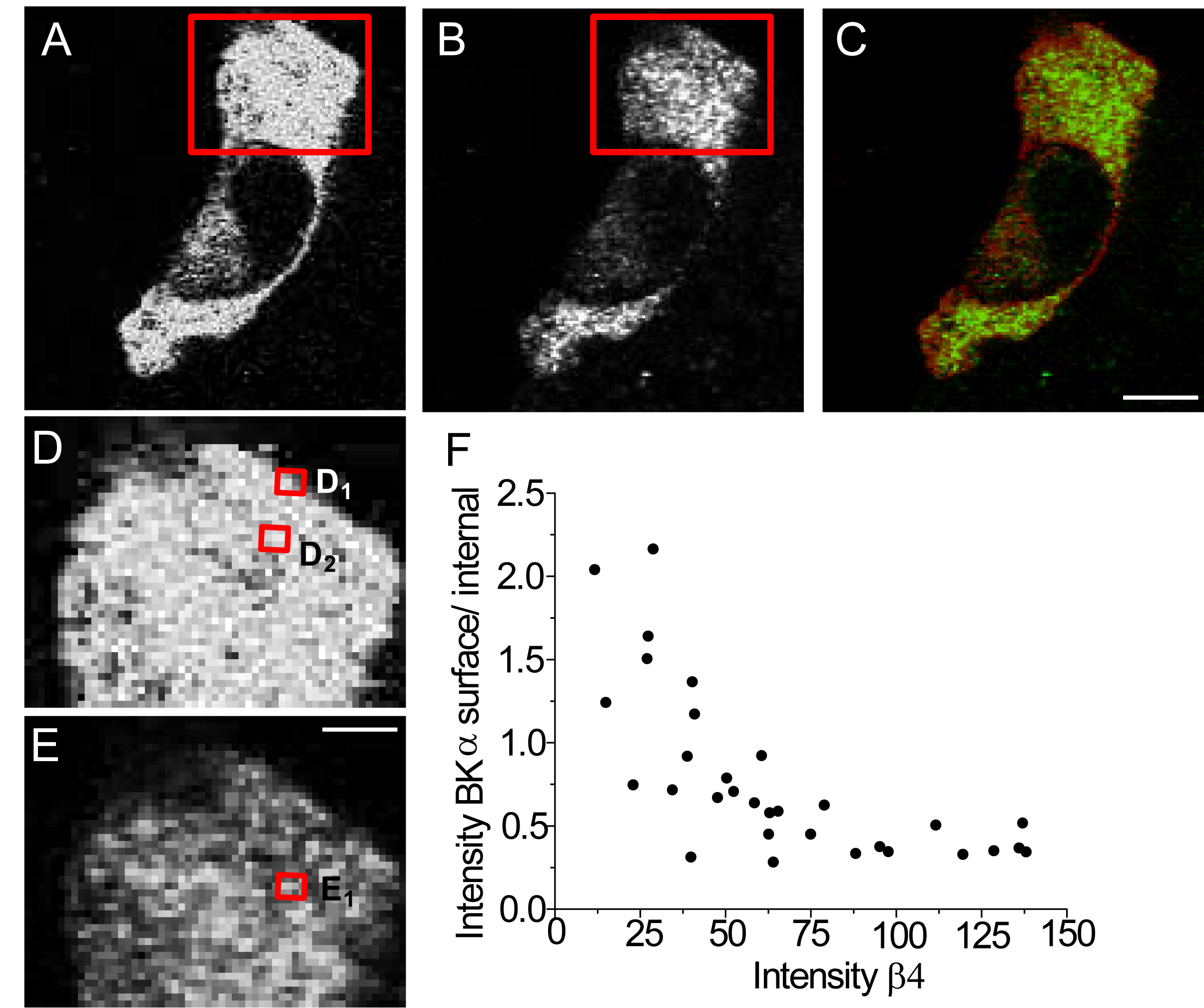

Supplement: Figure S3 — β4 reduces surface expression of BK channel α subunit. (A–C) Immunocytochemistry on fixed, permeabilized HEK-293 cell expressing FAP-BK α+β4 subunits. (A) Anti-BKα immunocytochemistry. (B) Anti-β4 immunocytochemistry for the same cell. (C) Merge of (A) in red and (B) in green. Scale bar = 10 µm (A–C). (D) Zoom of region within the red box in (A). The area within D1 and D2 are example ROIs used to calculate the intensity of surface (D1) and internal (D2) BKα expression, respectively, in a transfected cell. (E) Zoom of region within the red box in (B). The area within E1 is an example ROI used to calculate the intensity of β4 in a transfected cell. Scale bar = 5 µm (D–E). (F) Graph shows the mean BKα surface to internal ratio (i.e., D1/D2) to β4 expression (i.e., E1) for individual cells (30 ROIs per cell, n = 31 cells). (TIF) [file pone.0033429.s003.tif]
